# Supplementary figures and images for: Homothorax controls a binary Rhodopsin switch in Drosophila ocelli
Source: PLoS Genet. 2021 Jul 27;17(7):e1009460. doi: 10.1371/journal.pgen.1009460 (PMC8345863; doi:10.1371/journal.pgen.1009460)

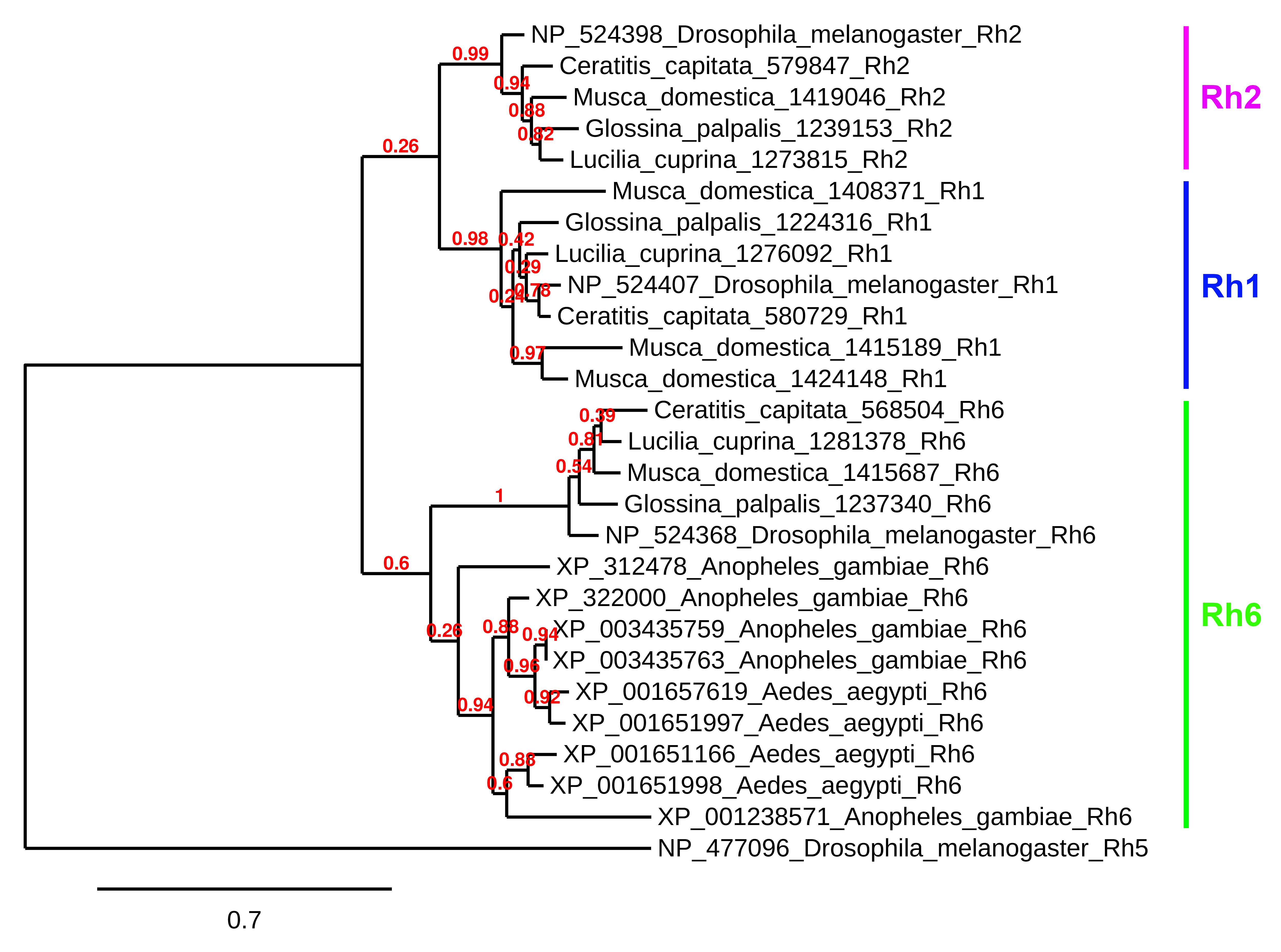

Supplement: S1 Fig — Phylogenetic tree by maximum likelihood method showing rh1, rh2 and rh6 duplications in different dipteran species, such as Drosophila melanogaster, Ceratitis capitata, Musca domestica, Glossina palpalis, Lucilia cuprina, Aedes aegypti and Anopheles gambiae. Opsin paralog groups were named according to Drosophila nomenclature. Amino acid sequences of Rhodopsins were used for making the phylogenetic tree and these sequences were available either in Feuda lab on bitbucket (https://bitbucket.org/Feuda-lab/opsin_diptera/src/master/) [33] or in NCBI (National Center for Biotechnology Information). The Phylogenetic tree was made (by using MUSCLE online tool phylogeny.fr) and Rh5 sequence from Drosophila was taken as outgroup. (TIF) [file pgen.1009460.s001.tif]

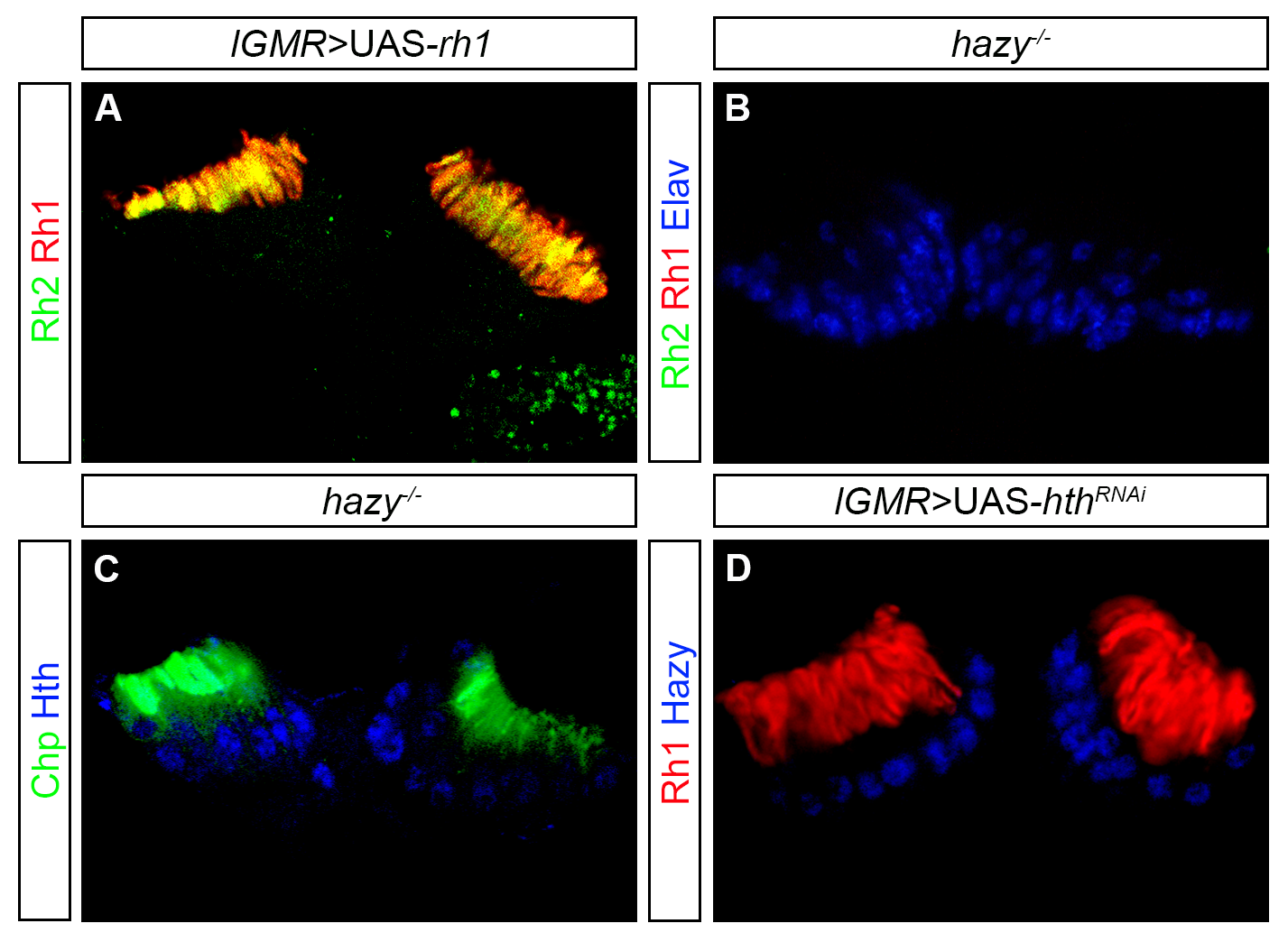

Supplement: S2 Fig — (A) Overexpression of rh1 in all PRs of ocelli by using pan-photoreceptor lGMR-Gal4. Antibody staining against Rh2 (green) and Rh1 (red) was performed to show that overexpression of rh1 does not inhibit Rh2 expression in ocelli. (B) Antibody staining to show expression of Rh2 (green) and Rh1 (red) in hazy-/- null mutant ocelli. Rh2 expression is lost in hazy-/- mutants but they don’t show ectopic Rh1 expression. (C) Antibody staining to show Hth expression (blue) in hazy-/- mutant ocelli, also marked by Chp (green). Hth expression is not affected in ocellar PRs in hazy-/- mutants. (D) Antibody staining to show Hazy expression (blue) in hth knockdown ocelli. Ocelli in hth knockdown are marked by staining against Rh1 (red). Hazy is expressed in hth knockdown ocelli that have lost Rh2 and gained Rh1 expression. (TIF) [file pgen.1009460.s002.tif]

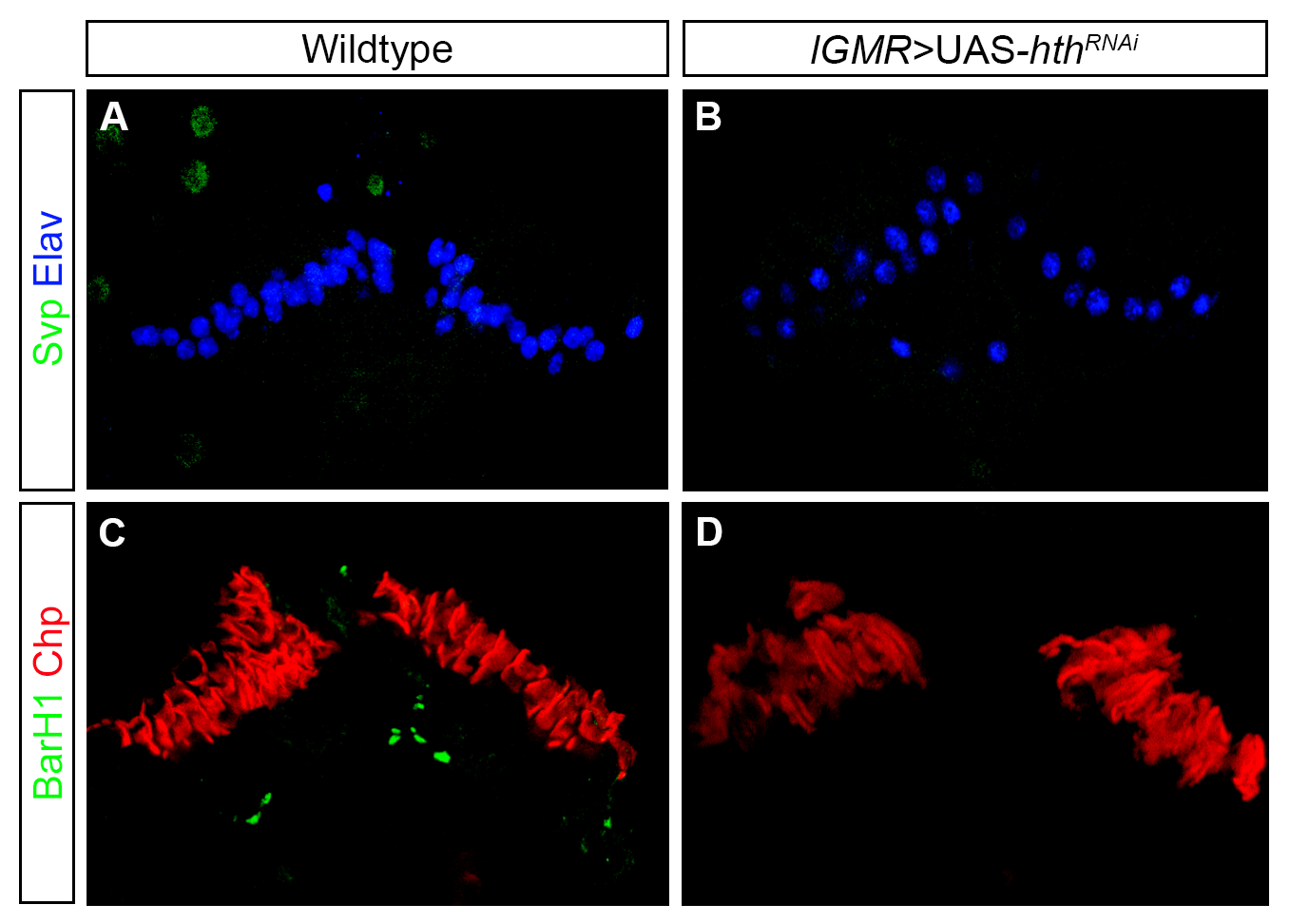

Supplement: S3 Fig — (A, B) Antibody staining against Svp (green) in the wildtype and in hth knockdown ocelli. Svp is normally expressed in R3/R4 and R1/R6 pairs of the developing retinal PRs [39] and not in wildtype ocelli (A). During the Rhodopsin switch by hth knockdown in ocelli, Svp is still not expressed in ocellar PRs (B). (C, D) Antibody staining against BarH1 (green) in the wildtype and in hth knockdown ocelli. BarH1 is normally expressed in R1/R6 pair of the developing retina [38] and not in wildtype ocelli (C). Upon Rhodopsin switch by hth knockdown, BarH1 is still not expressed in the ocellar PRs (D). Ocellar PRs are marked by antibody staining against Elav (blue, in A and B) and Chp (red, in C and D). (TIF) [file pgen.1009460.s003.tif]

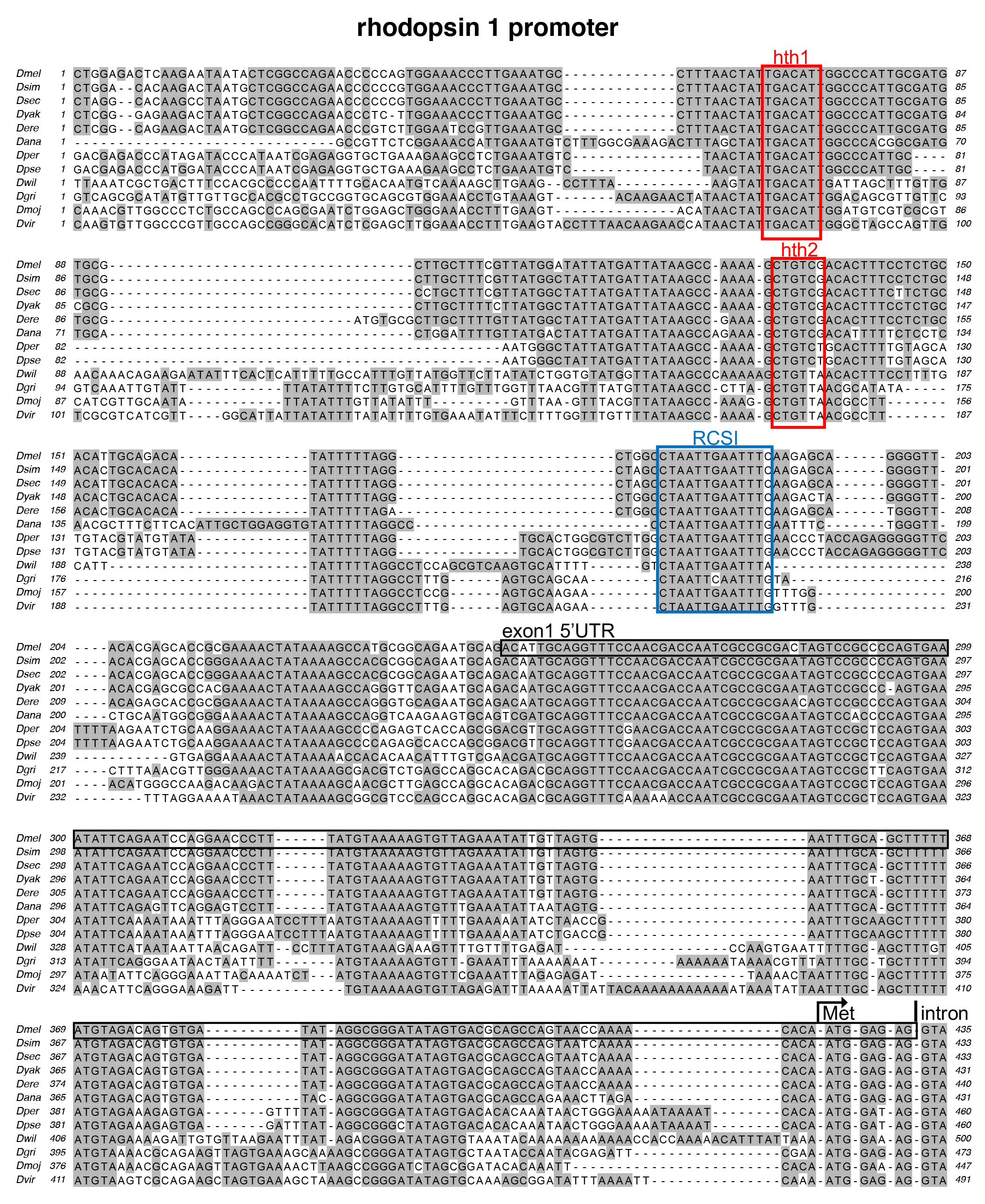

Supplement: S4 Fig — Conserved residues are depicted on a grey background. For the reporter constructs we cloned a 320 bp fragment ranging from nucleotides 2 to 321 of the D. melanogaster sequence in front of GFP. The two potential Hth binding sites are outlined in red. The RCSI is outlined in blue. The first Rh1 exon of D. melanogaster is outlined in black. In all twelve species the translation start (Met, arrow) is directly followed by an intron. Species used: D. melanogaster (Dmel), D. simulans (Dsim), D. sechellia (Dsec), D. yakuba (Dyak), D. erecta (Dere), D. ananassae (Dana), D. persimilis (Dper), D. pseudoobscura (Dpse), D. wilistoni (Dwil), D. grimshawi (Dgri), D. mojavensis (Dmoj), D. virilis (Dvir). (TIF) [file pgen.1009460.s004.tif]

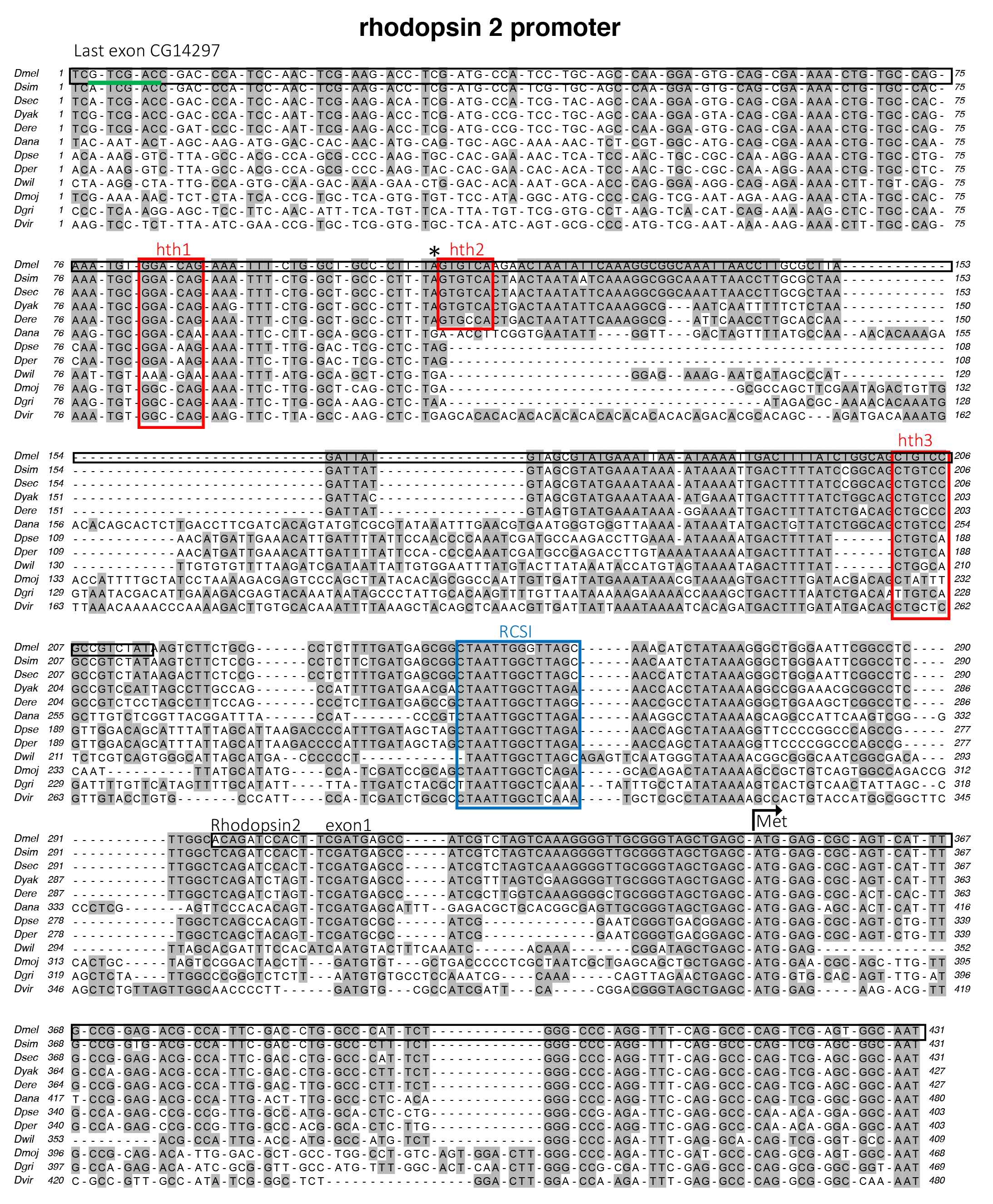

Supplement: S5 Fig — Conserved residues are depicted on a grey background. For the reporter constructs we cloned the 348 bp fragment of D. melanogaster from the endogenous Sal I restriction site (underlined in green) to the last nucleotide before the translation Start (Met, arrow) in front of GFP. This promoter sequence partially overlaps the last exon of the neighbouring gene CG14297. The three potential Hth binding sites are outlined in red. The first site is located within the coding sequence of CG14297. The second site which is only conserved within the melanogaster group (top five species) is located at the stop codon (asterisk) of CG14297, and the last site is located at the end of the 3’UTR. The RSCI (outlined in blue) is located within the 80 bp sequence between the end of the CG14297 3’UTR and the transcription start of Rhosopsin 2. The exons of D. melanogaster are outlined in black. Species used: D. melanogaster (Dmel), D. simulans (Dsim), D. sechellia (Dsec), D. yakuba (Dyak), D. erecta (Dere), D. ananassae (Dana), D. pseudoobscura (Dpse), D. persimilis (Dper), D. wilistoni (Dwil), D. mojavensis (Dmoj), D. grimshawi (Dgri), D. virilis (Dvir). (TIF) [file pgen.1009460.s005.tif]

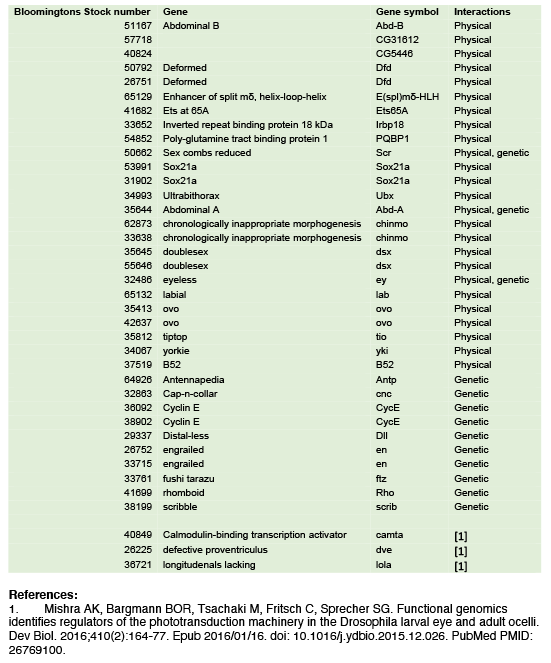

Supplement: S6 Fig — The table consists of candidates either known to have interactions with Hth (information from Flybase) or they have been previously found to regulate Rh2 expression [40]. For knockdown screening, flies were ordered from Bloomington’s Drosophila stock center and the stock numbers are listed in the table. (TIF) [file pgen.1009460.s006.tif]
