# Supplementary material for: Homothorax controls a binary Rhodopsin switch in Drosophila ocelli
Source: PLoS Genet. 2021 Jul 27;17(7):e1009460. doi: 10.1371/journal.pgen.1009460 (PMC8345863; doi:10.1371/journal.pgen.1009460)
Supplement: Attachment — Submitted filename: Rebuttal_Mishra.docx [file pgen.1009460.s007.docx]

**Reviewer #1:**

Evolutionary origin of the animal visual system is an important problem in biology. Evolutionary diversification of compound eye and ocelli of insects is a key process to solve this issue. In this study, the authors focused on the gene regulatory mechanism that specify the ocelli specific Rhodopsin2 (Rh2) expression and the compound eye specific Rh1 expression. They demonstrate that an evolutionarily conserved Homothorax (Hth) activates Rh2 transcription and represses Rh1in ocelli through the Hth-binding sites in the Rh1/2-promoters. The question is of general interest and the results are clearly demonstrated by a series of genetic experiments. However, the authors need to address the following concerns prior to the publication of the manuscript.

1. In Introduction, Results and Discussion sections, they indicate that Rh2 is UV-blue sensitive (418-506nm). According to the definition in line 111-113, it should be regarded as Blue-sensitive (or middle-wavelength according to line 294-297). However, in line 119-121, they indicate that Rh2 is long-wavelength (LW)-opsin (In Drosophila, PRs in the ocelli express the LW-opsin Rh2 that is sensitive to a wavelength range of 418-506 nm). I think the authors mean that Rh2 belongs to the LW-opsin group in the phylogenetic tree. This is confusing because the group of Rh1/2/6 contains rhodopsins of diverse wavelength. The groups in the phylogenetic tree should be named differently to avoid possible confusion.

**Thank you for the comment. We agree that it is a bit confusing to categorize *rhodopsins* based on their wavelength since the “long wavelength-opsin” group comprise opsins with a sensitivity range from violet to red. We have changed this in our result section (Differential expression of rhodopsins in the compound eye and ocelli) and in the discussion section (Rhodopsin duplication and role of Hth during ocelli diversification).**

2. The finding that hth activates Rh2 transcription and represses Rh1 in ocelli while it only activates Rh2 in compound eye is interesting. According to the results of Rh1 promoter-GFP and Rh2 promoter-GFP experiments, Hth activates Rh2 transcription and represses Rh1through the Hth binding sites. The authors should discuss why Hth does not repress Rh1 transcription in the compound eye.

**Thank you for the comment. It is indeed a good point. We believe that regulation of Rh1 and Rh2 in the ocelli and retina occurs via different ways. We provide evidence that Hth acts via the promoter of *rh1* and *rh2* and regulates Rh2 activation and Rh1 repression in the ocelli. The retina may lack an additional cofactor that may act together with Hth to regulate Rh1 expression in the ocelli. By performing a knockdown mini-screen, we identified Scrib and Ets65A as potential candidates that suppress Rh1 expression in the ocelli. However, a complete analysis should be done in order to find out their roles in regulating Rh1 expression. We have explained this in our manuscript in the result section (Role of Scrib and Ets65A as potential repressors of Rh1 in the ocelli) and in the discussion section (Genetic and molecular analysis of Hth in the ocelli during PR development).**

3. In Figs 1C-E, 2A-B and the other similar figures, the authors should explain why Rhodopsin expression does not overlap with nuclear signals such as Elav.

**Thank you for pointing this out. Since Rhodopsins are localized to the rhabdomere we do not detect overlap with nuclear Elav staining. We have now added a corresponding explanation to the legend of Figure 1.**

4. Line 204, 'mutating the’.

**Thank you. We have corrected the sentence.**

**Reviewer #2:**

The manuscript by Mishra and colleagues addresses the interesting questions of how rhodopsin genes are restricted to individual visual organs or classes of photoreceptors. The authors focus on the regulation of the Rh1 and Rh2 rhodopsin genes in the fruit fly Drosophila melanogaster. These two rhodopsin genes arose from a duplication event with Rh1 being expressed in the compound eye and Rh2 being expressed in the ocelli. The authors show convincingly that the homeodomain transcription factor Homothorax (Hth) is expressed in the ocelli where it activates expression of Rh2 and suppresses expression of Rh1. They go on to show that forced expression of Hth in the compound eye activates Rh2. Together the data suggest that Hth regulates a binary switch that maintains Rh2 expression in the ocellar photoreceptors.

The paper was a pleasure to read and I have only a couple of small suggestions for the authors.

**Thank you.**

1. Since Hth is known to interact with Extradenticle it might be interested if the authors could remove Exd using RNAi and see if Rh2 expression is turned off in the ocelli.

**Thank you for the comment. We have performed additional experiments addressing the involvement of Exd in the regulation of Rh2 in the ocelli. We could indeed show that also Exd is required for Rh2 expression. We included this data in Figure 2 and also in the manuscript in the result section (Homothorax regulates a binary rhodopsin switch in the ocelli).**

2. The authors note that Hth physically interacts with Engrailed to repress target gene expression. I think the authors should downregulated En with RNAi and see if Rh1 expression is activated in the ocelli.

**It is indeed a good point. We have downregulated *en* by knocking it down using two independent RNAi lines with *lGMR*-Gal4 in the ocelli. However, we could not find any phenotype in the ocelli and Rh2 expression was comparable to the wildtype. Therefore, we have not included these experiments in the manuscript. Moreover since we cannot provide experimental data on a putative function of engrailed in the ocelli we have removed this section from the discussion.**

3. The authors could also do a quick mini-screen of factors that are known to physically interact with Hth – I believe that information is listed within Flybase. The authors could use RNAi lines (where available) to knock them down with lGMR-GAL4 and assay for Rh1 and Rh2 expression.

**This is indeed a good point. We performed a RNAi mini-screen of candidates that are either known to regulate Rh2 expression (Mishra et al., 2016) or known to interact with Hth (listed in Flybase; Figure S6). We found that knockdown of those candidates that were previously shown to regulate Rh2 expression, a gain of Rh1 was not observed in the ocellar PRs. We found that knockdown of Scrib and Ets65A (potential interactors of Hth) derepresses Rh1 expression in the ocelli, without affecting Rh2 expression. We have included the data in Figure 6 and also in the manuscript in the results section (Role of Scrib and Ets65A as potential repressors of Rh1 in the ocelli) as well as in the discussion (Genetic and molecular analysis of Hth in the ocelli during PR development).**

Otherwise, the paper is really well written and the data within the figures are beautiful.

**Thank you!**

**Reviewer #3:**

In Drosophila, the rhodopsin Rh1 is expressed in the outer PR in the retina, while Rh2 is expressed in the ocelli. In this manuscript, the authors showed that the homeodomain protein Hth is expressed in the ocelli and induces rh2 transcription and suppresses rh1 transcription. The major results are (1) knockdown of Hth induced Rh1 (and rh1-lacZ) and eliminated Rh2 (and rh2-lacZ) in ocelli, (2) mutating the putative Hth-binding sites in the rh1 and rh2 minimal promoters eliminated rh2(hth_mut)-GFP expression and induced rh1(hth_mut)-GFP expression in ocelli. The effect of Hth is not through change in cell identity and not through regulation between rh1 and rh2. Therefore, it is concluded that hth controls a binary rhodopsin switch in ocelli.

In the retina, hth is not expressed. Ectopic expression of hth cell-autonomously induced Rh2 expression only in the outer PRs. However, Rh1 was not suppressed. Therefore, the “binary switch” does not operate in the retina.

The data are clean and nice. The results are clear cut. The finding that the choice of Rh1 and Rh2 expression in ocelli depends on Hth is nice but not so novel as Hth is known to regulate rh3 expression in the inner PRs in the dorsal rim area (DRA) of the compound eye. The novelty is only Hth exerts opposite transcriptional effect on the two genes. But this again is not novel if the two genes were not functionally related. The regulation by Hth on the two rhodopsin gens seems independent of each other. The evolutionary implication that Hth is involved in rhodopsin diversification needs more evidence.

Comments:
1. For the phylogenetic analysis of rhodopsin genes (Fig. 1B), the authors should expand their analysis to include rhodopsin genes from different representative branches of arthropods, perhaps even beyond the arthropods. Many genome sequences are now available.

**This is indeed a good point. As suggested we have now extended our phylogenetic analysis of long wavelength-opsins in different dipteran species and included it in Figure S1 and also in the manuscript in the result section (Differential expression of *rhodopsins* in the compound eye and ocelli).** **We provide evidence that the duplication events occurred in the lineage leading to higher Diptera (Rh2 being absent in *Anopheles* or *Aedes*, but present in *Musca* or *Lucilia*; as reported by the Feuda lab). Since Rh2 is absent in other insects (beyond Dipterans) an analysis in other arthropods or beyond would not provide significant information.**

2. The conclusion that Hth directly regulates rh1 and rh2 transcription is based on mutating the putative Hth binding sites in these promoters. The authors need to add experiments to show the direct binding of Hth to these binding sites.

**We agree that such experiments would strengthen our hypothesis that Hth directly regulates the expression of rh1 and rh2 by binding to the sites within their promoters. We have performed Electro-Mobility-Shift-Assays (EMSAs) on oligos comprising the wildtype or mutated versions of the five candidate Hth binding sites using an in vitro translated Hth protein. Unfortunately, we did not observe any bandshifts with any of the wildtype oligos (nor with the mutated ones).**

**It was previously shown that binding of Hth to DNA in EMSAs requires the presence of co-factors like Exd [Ryoo et al (1999) Development 126:5137-48; Gebelein et al (2004) Nature 432:653-659].We therefore reason that Hth does not bind alone, but rather that Hth/Exd is required for the rhodopsin regulation. We have now included these references and the point in the discussion section.**

3. The flp-out-clones with Hth expression were analyzed in the retina. Similarly, hth LOF clones should be performed in the ocelli to test whether the effects in ocelli on rh1 and rh2 expression is also cell-autonomous.

**This is an intersting point. We have performed this experiment, by inducing a heat-shock for Hth loss-of-function (LOF) clones at the same period as we did it for Hth gain-of-function (GOF) clones in the retina. Unfortunately, we did not observe either a loss of Rh2 or a gain of Rh1 in the clones in ocelli. There are a few possible reason why this experiment did not work: We reasoned that developmental timing of induction, protein perdurance may be critical for the phenotype. Therefore, we have not included this experiment in our manuscript.**

4. Based on the authors’ findings, Hth directly induces rh2 transcription but suppresses rh1 transcription. How to explain the opposite transcriptional regulation? Hth has been shown to be a transcriptional activator (Inbal et al., 2001). It can also participate in a transcriptional repressor complex (e.g. En-Hth-Exd; Kobayashi et al., 2003; Fujioka et al., 2012). The authors made some discussions, but did not pursue an answer. Since the rh1 and rh2 minimal promoter fragments can reflect the endogenous regulation, their sequence should be analyzed. Does the rh1 promoter also contains binding sites for repressor protein? Such binding site may be absent in the Rh2 promoter.

**Thank you for raising this point. We have now performed an analysis on the upstream sequence of both Rh1 and Rh2 and added them in two supplementary figures showing alignments of the rh1 and rh2 promoter sequences, respectively (Figure S4 and S5). Our analysis shows that indeed the Hth binding sites are conserved in closely related species. However, we could not observe clearly homologous repressor sites that are only present in one enhancer versus the other.**

5. In ocelli, Hth directly represses Rh1 and activates Rh2 transcriptionally. However, in the retina outer PRs, ectopic Hth can induce Rh2 expression but not repress Rh1 expression. This may suggest that the transcriptional repression of Rh1 requires some cofactor, e.g. a transcriptional repressor that can interact with Hth. This may further suggest such transcriptional repressor is present in the ocelli but absent in the outer PR of the retina. Can the authors provide some insight in this issue? For example, is the putative repressor (based on the binding sites in Rh1 promoter) expressed in the ocelli but not in retina?

**Thank you for the comment. It is indeed a good point. We have performed a knockdown mini-screen and identified Scrib and Ets65A as potential factors that suppress Rh1 expression in the ocelli. Further analysis is required in order to find out their roles on how they regulate Rh1 expression. We have also explained this in our manuscript in the result section (Role of scrib and Ets65A as potential repressors of Rh1 in the ocelli) and also in the discussion (Genetic and molecular analysis of Hth in the ocelli during PR development)**

6. In addition to the phylogenetic analysis of the rhodopsin coding region (not clear whether only the ORF were compared), the rh1 and rh2 minimal promoter sequence should also be analyzed phylogenetically in additional insect (and arthropods, if possible).

**For the phylogenetic analysis only the coding sequences were used. We have now clarified this in the manuscript. The *rh1* and *rh2* minimal promoter sequences are too divergent to compare between the two paralogs. Even within the orthologous promoter sequences of other Drosophilids there is a high degree of variation. Since Rh1 and Rh2 are unique to higher Diptera, their regulatory sequences cannot be compared to other insects or arthropods.**

7. For readers not familiar with the fly visual system, it would be nice if the authors can use an image to show the orientation and mark the different parts of the visual system, so the readers can understand what to look at in the figures.

**Thank you for this suggestion. We have added a zoomed image of head of a fly (Figure 1A) that shows location of compound eyes and ocelli. We have also added whole mount adult brain staining with anti-Rh2 that is specifically expressed in the ocelli (Figure 1B). We further explained the orientation of the stainings and localization of the marker proteins in the figure legend of Figure 1**

8. The Rh1 results may also be interpreted as rh1 being a default expression for all PRs, but is suppressed by Hth in the ocelli and perhaps suppressed by some unknown factor in the inner PRs.

**Thank you for this comment. We have indeed mentioned in our manuscript that Rh1 would be interpreted as a default state of Rhodopsin in the ocelli and it is being transcriptionally repressed by Hth. For the inner PRs of the retina, we do not have a solid evidence of how and if Rh1 is repressed in inner PRs and therefore we cannot make a comment on Rh1 being the default state of Rhodopsin in the retina.**

9. I have some reservation on the use of “binary switch”, but I would not make a strong point.

**Thank you, we indeed agree that in the current manuscript we do not assess a “binary cell fate switch”, but rather an “binary Rhodopsin switch”; we would therefore prefer to maintain this notion.**
