# Supplementary material for: Homothorax controls a binary Rhodopsin switch in Drosophila ocelli
Source: PLoS Genet. 2021 Jul 27;17(7):e1009460. doi: 10.1371/journal.pgen.1009460 (PMC8345863; doi:10.1371/journal.pgen.1009460)
Supplement: Attachment — Submitted filename: Rebuttal_revision_Mishra et al., 2021_AKM_22.06.docx [file pgen.1009460.s008.docx]

Reviewer #1:

The paper was properly revised.

**Thank you so much.**

However, I still have a concern about the description of 'long wavelength-opsin'. I don't get what was changed in the revised result and discussion sections. If 'long wavelength-opsin' is preferred for the name the group of rhodopsins Rh1/2/6 due to the historical reason of this field, it should be mentioned in the text.

**We agree that the violet-sensing Rh2 should not be labeled as a long-wavelength opsin. This categorization is based on its phylogenetic relationship with Rh6 whose orthologs in many insect species detect light in the blue-green range in contrast to other insect opsins that respond to UV-light. However, the spectral sensitivity of Rh2 is shifted towards violet wavelength of light. To avoid this confusion, we have now decided to entirely remove this categorization and used the labels “clade I” for Rh3, Rh4, and Rh5 branch of the tree and “clade II” for Rh1, Rh2, and Rh6 branch and we have simply introduced Rh1, Rh2 and Rh6 based on their unique wavelength sensitivity.**

Reviewer #2: The authors have addressed all of my comments. I recommend publication in PLOS Genetics. I am looking forward to seeing the paper in print!

**Thank you so much.**

Reviewer #3: The revision has addressed all of my comments.

**Thank you so much.**
